# Histone H3K9 Methylation Is Differentially Modified in Odontogenic Cyst and Tumors

**DOI:** 10.1055/s-0044-1791681

**Published:** 2024-11-07

**Authors:** Ekarat Phattarataratip, Aroonwan Lam-ubol

**Affiliations:** 1 Department of Oral Pathology, Faculty of Dentistry, Chulalongkorn University, Bangkok, Thailand; 2 Department of Oral Surgery and Oral Medicine, Faculty of Dentistry, Srinakharinwirot University, Bangkok, Thailand

**Keywords:** H3K9Me3, histone modification, odontogenic keratocyst, adenomatoid odontogenic tumor, ameloblastoma

## Abstract

**Objectives** Histone modification in odontogenic lesions is mostly unexplored. Trimethylation of histone H3 at lysine residue 9 (H3K9Me3) has been studied in various pathologic conditions and showed biological significance promising for future therapeutic application. This study aimed to investigate the level and clinical relevance of the H3K9Me3 histone modification in odontogenic cysts and tumors.

**Materials and Methods** A total of 105 cases of odontogenic lesions, comprising 30 odontogenic keratocysts (OKCs), 30 adenomatoid odontogenic tumors (AOTs), 30 ameloblastomas, and 15 dental follicles, were included in the study. The paraffin-embedded tissues were immunohistochemically stained for H3K9Me3. Both the intensity and the distribution of staining were evaluated and calculated as H-score. The correlation between the H3K9Me3 expression and the clinical characteristics of each lesion was evaluated.

**Statistical Analysis** The Kruskal–Wallis test followed by Bonferroni's correction was performed to assess the differences in H-score among groups. In addition, Pearson's chi-squared test or Mann–Whitney *U* test was used to analyze potential factors that could affect protein expression.

**Results** The reduced enamel epithelium of the dental follicle showed uniformly strong H3K9Me3 expression. All odontogenic cysts and tumors examined demonstrated a significantly reduced H3K9Me3 level compared with dental follicles. The AOT showed the lowest H3K9Me3 level, followed by OKC and ameloblastoma. Its immunoreactivity was mainly localized in the basal and parabasal cells of OKC and the whorled/duct-like structures of AOT. Ameloblastoma exhibited marked variation in the H3K9Me3 level among cases. Notably, the upregulated H3K9Me3 was related to multilocularity of OKC and ameloblastoma.

**Conclusion** Histone H3K9 methylation is differentially expressed in odontogenic cysts and tumors. This epigenetic modification may contribute to the pathogenesis and aggressive behavior of odontogenic lesions.

## Introduction

Odontogenic cysts and tumors are heterogeneous diseases arising within the jawbones. They comprise lesions with diverse clinical manifestations and disease behavior. Ameloblastoma is the most common odontogenic tumor with a locally aggressive nature. It is believed to originate from the remnants of odontogenic epithelium, such as the rests of the dental lamina, enamel organ, and odontogenic cyst lining. Conventional ameloblastoma demonstrates an infiltrative growth pattern and can cause extensive bone expansion, perforation, displacement, and resorption of adjacent structures. 1 The treatment for ameloblastoma requires surgical resection with a free margin to minimize recurrence. 1 In contrast, the adenomatoid odontogenic tumor (AOT), believed to also stem from an enamel organ, is an indolent disease with a rare chance of recurrence even after incomplete removal. 2 Odontogenic keratocyst (OKC) is a common odontogenic cyst arising from the dental lamina with unique clinical behavior. It can present radiographically as unilocular or multilocular and be destructive with a high recurrence rate. 3 4 It has been reported that the multilocular OKC had an increased recurrence rate and showed more aggressive behavior than the unilocular OKC. 5 Due to its aggressive nature and associated gene mutation, OKC was previously classified as an odontogenic tumor by the World Health Organization (WHO) in 2005. Although it is grouped within the odontogenic cyst in the current WHO classification, 6 controversies remain regarding its biological nature.

The pathogenic mechanisms of odontogenic lesions are still unclear. Several studies have shown evidence supporting the role of genetic and epigenetic changes, including gene mutation and DNA methylation. 7 8 Ameloblastoma and AOT have been shown to commonly harbor the mutated genes within the MAPK/ERK pathway, particularly the *BRAF* and *KRAS* , respectively. 7 9 Distinctively, OKC commonly demonstrated *PATCH1* mutation, which is involved in the hedgehog pathway. 7 These findings are rather perplexing as ameloblastoma, and AOTs manifest marked differences in biological behavior but share a common genetic pathway, suggesting other pathogenic mechanisms may be involved.

The epigenetic mechanisms are tightly regulated and play a crucial part during odontogenesis. 10 11 The aberrant regulation of these processes could, therefore, be responsible for the evolvement of odontogenic lesions. The altered levels of methylated or acetylated histones had been reported in various pathologic conditions involving the oral and maxillofacial region, such as salivary gland tumors, 12 13 oral epithelial dysplasia, and squamous cell carcinoma. 14 However, studies of epigenetic changes in odontogenic lesions are scarce. Recent findings emphasized the potential roles of epigenetic regulation, including the DNA methylation and histone acetylation at the H3K9, in ameloblastoma pathogenesis. 15

The role of trimethylation of histone H3 at lysine residue 9 (H3K9Me3) has been studied in the context of normal cellular development as well as tumorigenesis. It has been shown to affect several cellular processes, such as apoptosis, autophagy, and DNA repair. 16 17 18 19 Aberrant H3K9Me3 expression has been observed in several tumor types, including acute myeloid leukemia, 20 liposarcoma, 21 salivary gland carcinoma, 12 13 and cholangiocarcinoma. 22 Moreover, in several cancers, these changes appeared to correlate with their aggressive nature and treatment resistance. 13 21 23 However, the role of H3K9Me3 in odontogenic lesions has never been examined.

The aim of this study was to investigate the levels of trimethylated H3K9 in OKC, AOT, and ameloblastoma compared with the normal odontogenic epithelium of dental follicles. The findings of this study could potentially shed some light on the pathogenic mechanisms of these diseases and could lead to a better understanding and management of these lesions.

## Materials and Methods

### Study Population and Sample Selection

This study received ethical approval from the Human Research Ethics Committee of the Faculty of Dentistry, Chulalongkorn University (approval number 112/2021; HREC-DCU 2021–116). The sample size calculation was performed using G*Power 3.1.9.4, based on a previous study. 15 However, due to the lack of prior studies on H3K9Me3, a preliminary investigation with 30 samples per tumor type was conducted, revealing statistically significant differences with 90% power at a significance level of 0.05. Subsequently, 30 samples per type of odontogenic lesions were included.

The inclusion criteria included paraffin-embedded tissue specimens of patients diagnosed with OKC, AOT, and conventional (solid/multicystic) ameloblastoma of the jaws from the Department of Oral Pathology, Faculty of Dentistry, Chulalongkorn University from 2013 to 2022. The 15 dental follicle specimens were used as a control group. The unicystic and peripheral ameloblastoma and cases with insufficient tissue for study purposes were excluded. Clinical information, including patient age, sex, lesional site, clinical size, clinical manifestation, and radiographic features, was obtained from pathology submission forms and the patient's record. Features indicating the aggressiveness of the lesion, including multilocularity and recurrence, were recorded. The hematoxylin and eosin–stained slides of each case were reviewed by oral and maxillofacial pathologists to confirm the diagnosis.

### Immunohistochemical Staining

The immunohistochemical staining for H3K9Me3 was performed on the paraffin-embedded tissue according to the manufacturer's recommendation. Briefly, the specimens were deparaffinized and transferred to gradient alcohol. The antigen retrieval process was conducted with citrate buffer pH 6.0. After the washing step, endogenous peroxidase activity and nonspecific protein binding were blocked by 3% hydrogen peroxide solution (ChemSupply, Gilmore, Australia) and 10% fetal bovine serum (Sigma-Aldrich, California, United States), respectively. The specimens were incubated in primary antibody for H3K9Me3 (rabbit polyclonal antihistone H3 [tri-methyl K9] antibody, clone ab8898, Abcam, Cambridge, UK) in a humidified chamber at 4°C overnight, followed by the biotinylated goat anti-rabbit secondary antibody (clone P0448, Agilent Dako, California, United States). Color development was done using horseradish peroxidase followed by 3,3′-diaminodbenzidine (DAB) substrate (Liquid DAB+ Substrate Chromogen System, K3468, Agilent Dako). Then, hematoxylin was used for counterstaining. Negative control sections were prepared using the isotype-matched antibody (clone ab37415, Abcam).

### Scoring and Evaluation

Two oral and maxillofacial pathologists blindly evaluated the immunohistochemically stained slides at both low (×100) and high (×200 and ×400) magnifications without the knowledge of clinical and radiographic features. The calibration between the two pathologists was performed, and the final scoring was done simultaneously upon agreement by both pathologists. Intraexaminer reliability was checked by reevaluating one randomly selected case for every 10 cases examined. Only the immunostaining localized to the nuclei was considered positive. The distribution of H3K9Me3 expression among various cell types was recorded. The percentage of positively stained cells and their staining intensity were assessed for the whole sections to determine the H3K9Me3 level using the Histo-score (H-score). 12 24 The staining intensity was graded as 0 (no staining), 1+ (weak staining), 2+ (moderate staining), and 3+ (strong staining). The H-score, ranging from 0 to 300, was calculated by multiplying the percentage of stained lesional cells and the intensity scores. In addition, the H-score levels of ameloblastoma were categorized as low (<150) and high (≥150) for further analysis of clinically relevant factors.

### Statistical Analysis

Statistical analysis was performed using IBM SPSS Statistics version 21. The Kruskal–Wallis test, followed by Bonferroni's correction was conducted to evaluate the differences in H-score among groups. Pearson's chi-squared test, Fisher's exact test (for categorical data), or Mann–Whitney *U* test (for continuous data) was used as appropriate to analyze potential factors that could affect the H3K9Me3 expression. A *p* -value less than 0.05 was considered statistically significant. GraphPad Prism version 10.2.3 was used to generate the plot.

## Results

### Characteristics of the Study Population

A total of 30 OKCs, 30 AOTs, 30 ameloblastomas, and 15 normal dental follicles were included in the study. The average age of the study population for OKC, AOT, ameloblastoma, and dental follicle was 32.57 ± 13.99, 19.54 ± 9.74, 37.45 ± 20.42, and 16.17 ± 5.44 years, respectively. All entities except for AOT were found more commonly in the mandible than in the maxilla. Radiographically, 100% of dental follicles and AOTs demonstrated unilocular radiolucency. The majority (73.3%) of OKCs in this study presented as unilocular radiolucency, while multilocular radiolucency was observed in 26.7% of cases. In contrast, ameloblastoma almost equally appeared as unilocular (46.7%) and multilocular (53.3%) radiolucency. Histopathologically, the plexiform subtype was the most common (50%), followed by follicular (40%) and 10% of acanthomatous and desmoplastic subtypes. These subtypes were classified based on the predominant subtype, considering that more than one histopathologic subtype was observed in each lesion. Detailed information about disease characteristics is shown in Table 1 .

**Table 1:** Demographic and clinical characteristics of the study population

Characteristics	Dental follicle	Odontogenic keratocyst	Adenomatoid odontogenic tumor	Ameloblastoma
**Sex,** ***n*** **(%)**				
Male	11 (73.3)	14 (46.7)	3 (10.0)	12 (40.0)
Female	4 (26.7)	16 (53.3)	27 (90.0)	18 (60.0)
Age (y), mean ± SD Range	16.17 ± 5.44 8–24	32.57 ± 13.99 13–62	19.54 ± 9.74 6–59	37.45 ± 20.42 10–82
**Location,** ***n*** **(%)**				
Maxilla	5 (33.3)	5 (16.7)	18 (60.0)	1 (3.3)
Mandible	10 (66.7)	25 (83.3)	12 (40.0)	29 (96.7)
**Size,** ***n*** **(%)**				
≤2 cm	100 (100.0)	11 (36.7)	10 (33.3)	5 (16.7)
> 2 cm	0 (0.0)	19 (63.3)	20 (66.7)	25 (83.3)
**Radiographic features,** ***n*** **(%)**				
Unilocular radiolucent	15 (100.0)	22 (73.3)	20 (66.7)	14 (46.7)
Multilocular radiolucent	0 (0.0)	8 (26.7)	0 (0.0)	16 (53.3)
Mixed radiolucent–radiopaque	0 (0.0)	0 (0.0)	10 (33.3)	0 (0.0)
**Recurrence,** ***n*** **(%)**				
Yes	0 (0.0)	8 (26.7)	0 (0.0)	5 (20.0)
No	15 (100.0)	22 (73.3)	100 (100.0)	25 (80.0)

### The H3K9Me3 Levels in OKC, AOT, and Ameloblastoma were Significantly Decreased Compared with Dental Follicle

**Fig. 1:**
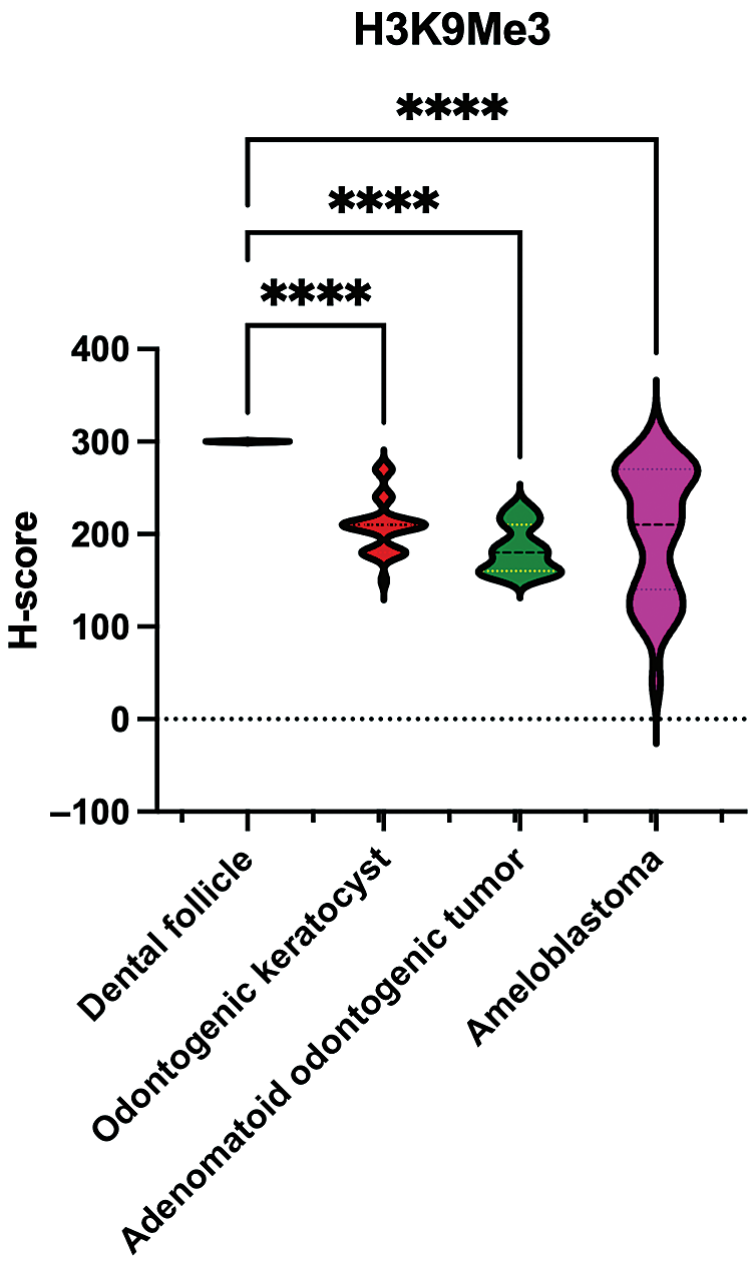
The H3K9Me3 levels in OKC, AOT, and ameloblastoma compared with the dental follicle. The statistical analysis was performed using the Kruskal–Wallis test followed by Bonferroni correction (**** *p* < 0.0001). AOT, adenomatoid odontogenic tumor; OKC, odontogenic keratocyst.

We observed the variably positive nuclear staining of H3K9Me3 within the epithelial cells of all odontogenic lesions examined. Notably, the dental follicle showed uniformly strong and diffuse nuclear expression of H3K9Me3 with a median H-score of 300. In contrast, the median H-scores for OKC, AOT, and ameloblastoma were 210, 180, and 210, respectively. Statistically, OKC, AOT, and ameloblastoma demonstrated significantly lower H3K9Me expression than the control dental follicle ( *p* < 0.0001; Fig. 1 ).

### The OKC, AOT, and Ameloblastoma Showed a Distinct H3K9Me3 Expression Pattern

Differing from that of the dental follicle, which showed uniformly strong H3K9Me3 expression in all layers of reduced enamel epithelium as well as the stromal odontogenic rests, odontogenic epithelial cells of OKC, AOT, and ameloblastoma demonstrated a relatively variable but unique expression pattern among different cell types.

**Fig. 2:**
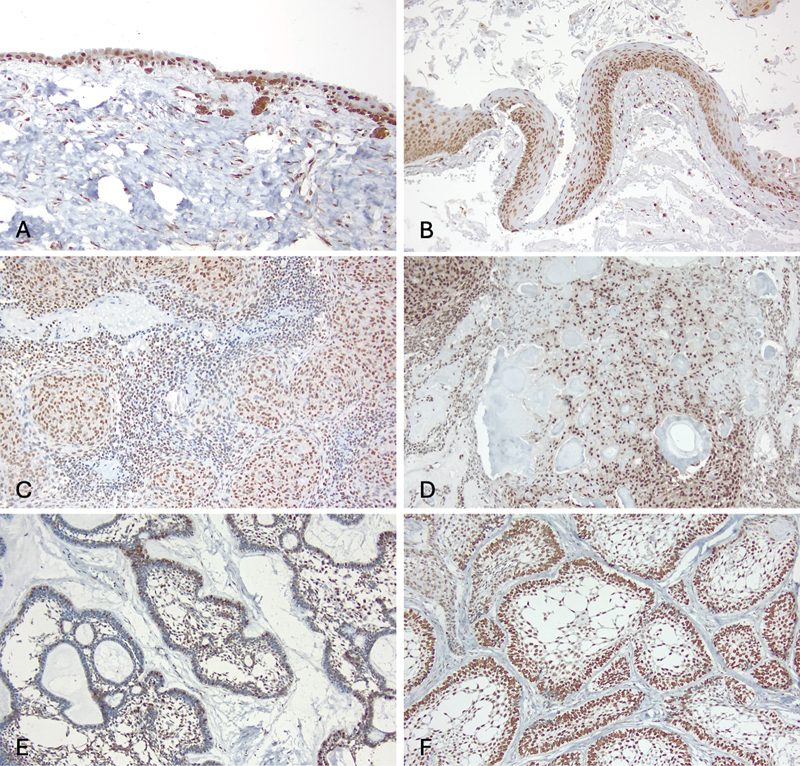
The H3K9Me3 immunoreactivity and cellular localization in ( **A** ) dental follicle, ( **B** ) OKC, ( **C** ) AOT, ( **D** ) CEOT-like area in AOT, ( **E** ) ameloblastoma with low expression, and ( **F** ) ameloblastoma with high expression (magnification ×100). AOT, adenomatoid odontogenic tumor; CEOT, calcifying epithelial odontogenic tumor; OKC, odontogenic keratocyst.

OKCs strongly expressed H3K9Me3 within the basal and parabasal portion of the cystic epithelial lining epithelium, while the superficial portion of the lining showed limited and variable staining. In AOTs, H3K9Me3 was primarily localized in the neoplastic cells forming whorled masses and duct-like structures, whereas those cells forming the anastomosing odontogenic epithelial strands exhibited relatively weak or no expression. The increased H3K9Me3 level was also observed within the calcifying epithelial odontogenic tumor (CEOT) like areas in selected AOT cases. Interestingly, in ameloblastoma, H3K9Me3 was variably expressed among cases in both the ameloblast-like and stellate reticulum–like cells. The levels and cellular distribution of H3K9Me3 are presented in Table 2 and Fig. 2 .

**Table 2:** Cellular distribution and levels of H3K9Me3 expression among odontogenic cysts and tumors

Odontogenic lesions	Cellular population	Staining intensity
Odontogenic keratocyst	Basal and parabasal cells Superficial cells and keratin	+++ +/−
Adenomatoid odontogenic tumor	Whorled masses Duct-like structures Odontogenic epithelial strands CEOT-like areas	+++ +++ +/− +++
Ameloblastoma	Ameloblast-like cells Stellate reticulum-like cells Squamous metaplasia	++/+ ++ ++/+ ++ +++
Dental follicle	Cuboidal lining epithelium Odontogenic rests	+++ +++

Abbreviation: CEOT-like areas, calcifying epithelial odontogenic tumor-like areas.

### The Upregulated H3K9Me3 in OKC and Ameloblastoma Was Significantly Correlated with Multilocularity

We then investigated the relationship between variable H3K9Me3 expression and clinical factors, including patient sex, location and size of the lesion, radiographic appearances, and the recurrence of odontogenic cysts and tumors. We found that the multilocular OKC significantly upregulated H3K9Me3, compared with unilocular cases ( *p* = 0.031). No correlation was noted in other clinical factors. The detailed analysis is shown in Table 3 .

**Table 3:** Factors affecting H3K9Me3 expression in OKC, AOT, and ameloblastoma

Characteristics	H-score, median (IQR)					
	Odontogenic keratocyst	*p* -value a	Adenomatoid odontogenic tumor	*p* -value a	Ameloblastoma	*p* -value a
**Sex**						
Male	210 (30)	0.423	210 (37.5)	0.516	225 (135)	0.484
Female	210 (15)		177.5 (28.75)		210 (125)	
**Location**						
Maxilla	210 (0)	0.787	180 (30)	0.245	270	0.452
Mandible	210 (30)		192.5 (56.25)		210 (130)	
**Size**						
≤2 cm	210 (0)	0.598	170 (46.25)	0.668	240 (60)	0.62
> 2 cm	210 (37.5)		180 (37.5)		210 (130)	
**Radiographic features**						
Unilocular radiolucent	210 (30)	0.031 b	180 (37.5)	0.769	175 (142.5)	0.077
Multilocular radiolucent	240 (45)		N/A		240 (95)	
Mixed radiolucent–radiopaque	N/A		172.5 (46.25)		N/A	
**Recurrence**						
Yes	210 (30)	0.298	N/A	–	270 (30)	0.136
No	210 (45)		180 (50)		210 (150)	

Abbreviations: AOT, adenomatoid odontogenic tumors; IQR, interquartile range; N/A, not applicable; OKC, odontogenic keratocysts.

a *p* -value as analyzed by Mann–Whitney *U* test.

b Statistically significant ( *p* < 0.05).

Because ameloblastoma showed marked variation in H-score among cases, we further categorized the H-score levels into low (H-score < 150) and high (H-score ≥ 150) expression groups. Interestingly, the analyses revealed a similar trend as noted in OKC. As shown in Table 4 , we found that the multilocular ameloblastomas showed significantly a greater H3K9Me3 expression than the unilocular subtype ( *p* = 0.032). Specifically, 66.7% of ameloblastomas with a high H3K9Me3 expression presented as multilocular radiolucency, whereas this feature was noted only in 22.2% of the low expression group. In addition, we noted that none of the ameloblastomas with low H3K9Me3 expression exhibited recurrence, whereas 23.8% of highly expressed ameloblastoma cases recurred ( Table 4 ). These data suggest that hypermethylation of H3K9 could be involved in the aggressive nature of ameloblastoma.

**Table 4:** Factors affecting the levels of H3K9Me3 expression in ameloblastoma

Characteristics	Levels of H3K9Me3, % of cases		
	Low	High	*p* -value a
**Sex**			
Male	44.4	36.4	0.489
Female	55.6	63.6	
**Location**			
Maxilla	0.0	4.5	0.71
Mandible	100.0	95.5	
**Size**			
≤2 cm	11.1	18.2	0.542
>2 cm	88.9	81.8	
**Radiographic features**			
Unilocular radiolucent	77.8	33.3	0.032 b
Multilocular radiolucent	22.2	66.7	
**Recurrence**			
Yes	0.0	23.8	0.143
No	100.0	76.2	

a *p* -value as analyzed by Fisher's exact test.

b Statistically significant ( *p* < 0.05).

## Discussion

We investigate the impact of histone H3 modification in common odontogenic cysts and tumors, including OKC, AOT, and ameloblastoma. To our knowledge, this is the first study to evaluate the level of trimethylated H3K9 in odontogenic lesions and its relationship with the clinical-pathologic data. We observe the variable expression levels among different cell types of these lesions. Remarkably, the significantly reduced H3K9Me3 is detected in OKC, AOT, and ameloblastoma, compared with the control normal odontogenic epithelium of the dental follicle. The analyses within the lesions reveal that the hypermethylated H3K9 is significantly correlated with the presence of multilocularity, the feature shown to be associated with higher recurrent rates of both OKC and ameloblastoma. 5 25

The role of epigenetic regulation, including DNA methylation and histone modifications, in the pathogenesis of odontogenic lesions has been scarcely studied. 7 do Amaral-Silva et al reported that DNA methylation could be involved in its pathogenesis in ameloblastoma. However, histone modification, specifically the H3K9Ac, demonstrated limited potential as its expression was not different among ameloblastoma and normal dental follicles. 15 Interestingly, ameloblastic carcinoma showed increased levels of DNA methyl transferases and H3K9Ac, suggesting that the altered epigenetic regulation could be involved in the increased aggressiveness or malignant potential of these lesions. 15 Nevertheless, due to the rarity of the lesion, only six ameloblastic carcinoma cases were included in their study. The polycomb repressive complex proteins have been shown to regulate cellular differentiation epigenetically via histone H3K27Me3 modification. 26 Their expression was variably expressed in ameloblastoma and OKC. 27 However, no expression in AOT or clinical correlation was examined.

Although the change in methylated H3K9 has not been previously explored in odontogenic cysts and tumors, a study in tooth germ reports that the four H3K9 methyltransferases (G9A, SETDB1, SUV39H1, and PRDM2) are highly expressed in the odontogenic mesenchyme in mice. 28 In addition, Gopinathan et al observed the dynamic histone modification response in dental follicles during the mineralization induction phase and the enrichment of H3K9Me3 at the promoters of late mineralization genes (i.e., *OSX* , *IBSP* , and *BGLAP* ) in both the dental pulp and dental follicle progenitors. 29 These data indicate the contribution of H3K9 methylation during odontogenesis, and its dysregulation could potentially underlie the pathogenesis of odontogenic lesions.

We observed reduced H3K9Me3 expression in OKC, AOT, and ameloblastoma compared with dental follicles. This could be because these odontogenic lesions arise from other cell types, such as the dental lamina or enamel organs. In addition, the odontogenic epithelium in these cystic and neoplastic conditions is likely in an active/proliferative stage of development. In contrast, the odontogenic epithelial remnants in dental follicles exist in a relatively mature, quiescent, or inactive phase of tooth development. Therefore, H3K9Me3 regulation in these lesions could differ entirely from dental follicles. However, within the same entity, we found that H3K9Me3 is differentially expressed, and its altered methylation level could affect the aggressive nature of these lesions.

The histone H3K9 methylation level and the associated methylation enzymes SETDB1 and SUV39H1/2 have been widely investigated in human neoplasms. 30 31 The overexpression of H3K9Me3 was correlated with the aggressiveness of various malignancies, including prostate cancer, 32 renal cell carcinoma, 33 34 hepatocellular carcinoma, 35 liposarcoma, 21 and colorectal cancer. 36 The dysregulation of SETDB1 was associated with the progression and/or resistance to treatment in cancers of the nasopharynx, 37 breast, 38 acute lymphoblastic leukemia, 39 stomach, 40 colon, 41 skin, and lung. 42 SUV39H1/2 was also shown to be involved in the pathogenesis of renal cell carcinoma, 43 prostate cancer, 32 and malignant glioma. 44

Our findings advocate that H3K9Me3 may be involved in the pathogenesis and aggressiveness of ameloblastoma, AOT, and OKC. While the specific mechanisms underlying this observation are still unclear and may differ among various entities, H3K9Me3 is known for regulating heterochromatin in gene expression silencing. 19 45 Moreover, it has been shown to crosstalk with DNA methylation to control gene expression. 46 Functionally, H3K9Me3 could modulate various cellular processes, such as apoptosis, autophagy, development, and DNA repair. 16 17 18 19

Several mechanisms underlying the contribution of H3K9Me3 in promoting aggressive tumor characteristics have been proposed, and these may vary among tumor types. For example, H3K9Me3 was shown to decrease E-cadherin expression and thereby promote epithelial-mesenchymal transition in lung cancer. 47 In colon cancer, it could silence Fas expression, inhibiting apoptosis. 36 In melanoma, SETDB1 activates thrombospondin-1 and is responsible for maintaining tumorigenic function, 48 whereas it abrogates the p53, Bak, Bax, and Bim activity in pancreatic cancer. 23 49

The pathogenesis of odontogenic cysts and tumors is poorly understood. Our previous studies demonstrate that claudin-1, the tight junction protein known for its role in oncogenesis, 50 51 is variably expressed in different cell types of odontogenic lesions. Reverse to what we observed of the H3K9Me3 expression pattern in the present study, claudin-1 shows diminished expression in ameloblast-like cells, ductal/whorled structures, and basal/parabasal cells of ameloblastoma, AOT and OKC, respectively. 52 53 These data appear to correspond to the finding that the expression of claudin-1 is predominantly regulated by H3K9 methylation. 54 Notably, the reduced claudin-1 expression is associated with the increased recurrence of ameloblastoma. 52 Therefore, it is possible that the enrichment of H3K9Me3 at the promoter region of the claudin-1 gene could lead to its transcriptional silencing and confer the more aggressive phenotype of ameloblastoma.

In addition, H3K9Me3 may exert its effects through the epigenetic crosstalk with selected genetic pathways responsible for the development and progress of these lesions. In melanoma with *BRAF* mutation, the upregulated H3K9Me3 level was reported to be associated with increased treatment resistance. 42 Moreover, H3K9Me3, as well as its associated methylation enzymes (SUV39H2 and SETDB1), was shown to regulate gene expression in the hedgehog and Wnt/B-catenin signaling pathways. 22 44 Since these genetic alterations are also detected in odontogenic lesions, including ameloblastoma, AOT, and OKC, it is likely that these crosstalks between genetic and epigenetic mechanisms exist and deserve further investigation. These future studies may also explain the differences in biological behavior between ameloblastoma and AOT despite sharing a common genetic pathway.

Our study had certain limitations resulting from the heterogeneity and uncommon nature of these lesions. In addition, the retrospective nature of this study precluded the ability to obtain long-term clinical data. To minimize heterogeneity, we specifically included the conventional (solid/multicystic) ameloblastoma since this subtype behaves locally aggressively and poses significant disease morbidity. Even though the conventional ameloblastoma cases in our study comprised different histopathologic patterns, including follicular, plexiform, and acanthomatous variants, they were shown to not correlate with the clinical behavior of ameloblastomas or impact on patient prognosis. 55 In addition, due to the limited numbers of recurrent ameloblastoma in our study, the precise contribution of H3K9Me3 to tumor recurrence has not been determined. Moreover, recurrence may also be influenced by clinical factors, such as treatment modality, locations or sizes of lesions, and patient conditions. 56 Future prospective studies with a larger population will be required to investigate this aspect. Nevertheless, our results revealed high statistical discrimination power with the number of cases examined, and the clinical correlation with the altered histone modification was performed. Future experiments to investigate SETDB1 and SUVH31 expressions in odontogenic lesions may be beneficial as these enzymes are now in the clinical trials for various cancers. 31

## Conclusion

Aberrant histone H3K9 methylation is detected in odontogenic cysts and tumors. Its expression varied among different cell types and lesions. These changes in methylated histone H3 may contribute to the development of odontogenic lesions and the aggressive characteristics of OKC and ameloblastoma. The findings provide more understanding of the pathogenesis of odontogenic lesions and the potential development of treatment strategies.
